# Radiographic assessment and orthodontic intervention effects on orthodontically induced root resorption: a systematic review and meta-analysis of clinical trials

**DOI:** 10.2340/aos.v85.46818

**Published:** 2026-09-15

**Authors:** Khalid Ayidh Alqahtani, Hamod Alqahtani, Abdullah Saad Alqahtani, Mahmud Uz Zaman, Wajdi A. Mohammed (Bin), Saleh Alhindi, Nasser Raqe Alqhtani

**Affiliations:** a Department of Oral and Maxillofacial Surgery and Diagnostic Sciences, College of Dentistry, Prince Sattam Bin Abdulaziz University, Al-kharj, Saudi Arabia; b Department of Prosthetic Dental Sciences, College of Dentistry, Prince Sattam Bin Abdulaziz University, Al-kharj, Saudi Arabia; c Department of Preventive Dental Sciences, College of Dentistry, Prince Sattam Bin Abdulaziz University, Al-kharj, Saudi Arabia; d Department of Oral Medicine and Diagnostic Sciences, College of Dentistry, King Saud University, Riyadh, Saudi Arabia; e Department of Conservative Dental Sciences, College of Dentistry, Prince Sattam Bin Abdulaziz University, Al-kharj, Saudi Arabia

**Keywords:** Radiographic methods, root resorption, orthodontic treatment, detection accuracy, systematic review

## Abstract

The relative contribution of radiographic methods and characteristics of the orthodontic intervention to orthodontically induced root resorption (OIRR) remains unknown. The aims of this systematic review and meta-analysis were to (1) estimate the pooled OIRR effect across orthodontic intervention versus comparator contrasts, (2) compare pooled estimates by radiographic method (2D [two-dimensional] vs. 3D/CBCT [three-dimensional/cone-beam computed tomography]), and (3) explore whether force mechanics (intrusive versus nonintrusive) modified OIRR magnitude.

Seven randomized controlled trials and one prospective study (January 2010–October 2025) were included. Only OIRR was the outcome, reported as correlation coefficients (*r*). The primary analysis combined within-study intervention-versus-comparator estimates. Subgroup analysis of 2D versus 3D/CBCT imaging was prespecified, whereas post-hoc analysis of intrusive versus nonintrusive mechanics was performed.

The pooled analysis for the primary outcome showed a small, nonsignificant OIRR effect (*r* = 0.07; 95% confidence interval [CI]: −0.12 to 0.27; *p* = 0.372) with high heterogeneity (*I*^2^ = 84.0%). Radiographic method did not change the pooled estimates significantly (*p* = 0.331). Force-mechanics analysis showed that intrusive mechanics was related to significantly higher root resorption than nonintrusive mechanics (*r* = 0.40; 95% CI = 0.15 to 0.65 versus *r* = −0.03; 95% CI = −0.16 to 0.10; *p* < 0.001). This accounted for 87.1% of the between-study variance.

The average orthodontic intervention effect on OIRR was small and not significant; however, the OIRR magnitude was strongly affected by force mechanics, particularly by intrusive forces. There was no significant difference in pooled estimates by radiographic method; however, 3D/CBCT provides superior volumetric quantification and should be used judiciously according ALARA (as low as reasonably achievable) principles.

## Introduction

Root resorption is a known, frequently inevitable, iatrogenic effect of orthodontic movement of the teeth, which involves loss of dental hard tissues at the apex of the root or along the root surface. Root resorption induced by orthodontics (OIRR) is a highly variable phenomenon with almost all patients and as many as 91% of teeth showing some amount of root shortening following the procedure, but the most severe cases are rather uncommon [1–3]. OIRR also has a multifactorial etiology and includes patient-related factors (genetics, age, gender, tooth type, root morphology, and history of trauma) and treatment-related factors (appliance type, length of treatment, magnitude of force, and extraction protocols) [1, 3, 4].

The detection of root resorption at a relatively early stage, along with timely and correct diagnosis, is very important, as the symptoms of the disease do not manifest at all until later in life, and radiography becomes the main element in diagnostics [1, 5, 6]. The traditional two-dimensional (2D) radiology techniques, including the periapical and panoramic radiographs, have long been used for monitoring root resorption as they are readily available, relatively cheap, and the amount of radiation to which they expose the patient is relatively low [5–7]. These methods, however, are constrained to problems of superimposition, magnification errors, and inability to recognize resorption at buccal or lingual surfaces, which usually leads to low estimation of the actual extent of resorption [7–9]. It is demonstrated that panoramic radiographs can greatly underreport root resorption in comparison to more sophisticated imaging procedures [6, 10, 11].

Recently, cone-beam computed tomography (CBCT) has transformed the evaluation of root resorption because it offers three-dimensional (3D) images, which make the measurement of root length and volume more accurate and consistent [1, 2, 8, 9]. Kara Boulad et al. demonstrated CBCT’s practical utility for quantifying apical root resorption and alveolar bone changes after orthodontic treatment, reinforcing the modality’s clinical relevance [12]. CBCT is more sensitive and specific in not only identifying the presence and severity of root resorption but also in poorly defined cases of 2D imaging [2, 5, 8, 10]. The most recent meta-analyses and systematic reviews prove that CBCT is a valid instrument to assess OIRR, but the typical value of resorption measured is not clinically significant in most instances [1, 5, 8]. However, application of CBCT should be countered against its relatively high cost and excessive radiation exposure, which makes it a better complementary diagnostic device, but not the one that should be used regularly as a monitoring procedure [5, 6, 9].

Future technologies, like automated CBCT image segmentation using deep learning, are also improving the quality and reliability of root resorption measurement and allow the volumetric analysis and better clinical decision-making [2, 13, 14]. Although progress has been made, high-quality and prospective studies have been emphasized in the literature toestablish the risk factors and improve the imaging protocols [1, 8, 15].

To conclude, although traditional radiographic tools are still the most important to use when the presence of root resorption is to be detected regularly during the course of the orthodontic treatment, CBCT has better diagnostic accuracy and should be used in situations where accurate measurements are very important in terms of treatment planning or adjustment [1, 5, 6, 8]. The balance between diagnostic benefit and patient safety in the radiographic assessment of OIRR is still being enhanced by ongoing research and technological innovation.

Therefore, this systematic review and meta-analysis synthesizes clinical-trial evidence to address the following aligned aims: (1) to estimate the pooled OIRR effect across orthodontic intervention-versus-comparator contrasts; (2) to compare pooled estimates according to radiographic assessment method, specifically 2D imaging (periapical or panoramic radiography) versus 3D imaging (CBCT); and (3) to explore, through a post-hoc subgroup analysis, whether orthodontic force mechanics classified as intrusive versus nonintrusive modify the magnitude of OIRR.

## Methods

### Study design

The review was conducted in accordance with the Preferred Reporting Items for Systematic Reviews and Meta-Analyses (PRISMA) guidelines [16] and followed the recommendations of the Cochrane Handbook for Systematic Reviews of Interventions [17]. The review was prospectively registered under PROSPERO number CRD420251245978. The review protocol was prepared prior to study commencement and is accessible via the PROSPERO record at: https://www.crd.york.ac.uk/PROSPERO/view/CRD420251245978 .

### Literature search strategy

A comprehensive and exhaustive electronic search was performed across multiple databases, including PubMed/MEDLINE, Embase, Scopus, Web of Science, Cochrane Central Register of Controlled Trials, and additional sources such as ClinicalTrials.gov and OpenGrey. The search strategy combined keywords and controlled vocabulary (e.g. MeSH, Emtree) related to ‘root resorption’, ‘orthodontic treatment’, ‘radiographic methods’, and ‘CBCT’. Boolean operators and truncation were used to maximize sensitivity. The search was restricted to English-language articles published between January 2010 and October 2025 to capture contemporary evidence reflecting modern orthodontic techniques and CBCT technology. Reference lists of included studies and relevant reviews were also manually screened to identify additional eligible articles (Table 1).

**Table 1:** Detailed search strategies and filters for each database.

Database	Search query components	Applied filters	Syntax/modifiers
PubMed/MEDLINE	(“root resorption” OR “external apical root resorption”) AND (“orthodontic” OR “CBCT”)	Humans, English, 2010–October 2025	MeSH terms, Boolean operators, truncation (*)
Embase	(‘root resorption’/exp OR ‘EARR’) AND (‘orthodontic treatment’/exp OR ‘CBCT’)	Humans, English, 2010–October 2025	Emtree terms, Boolean operators, wildcards ($)
Scopus	TITLE-ABS-KEY(“root resorption” AND “orthodontic” AND “radiograph*”)	English, 2010–October 2025	Field tags, Boolean operators, and truncation (*)
Web of Science	TS=(“root resorption” AND “orthodontic” AND (“CBCT” OR “radiograph*”))	English, 2010–October 2025	Topic search (TS), Boolean operators, truncation
Cochrane Library	(“root resorption” AND “orthodontic” AND “radiograph*”)	Trials, Reviews, 2010–October 2025	Boolean operators, phrase searching (“”)
OpenGrey	“root resorption” AND “orthodontic” AND “radiograph*”	None	Phrase searching, Boolean operators

In addition to electronic searches, manual screening of reference lists from included articles and relevant reviews was performed to identify further eligible studies. Two reviewers independently conducted all stages of study selection and data extraction. Disagreements were resolved through discussion, and if consensus was not reached, a third reviewer was consulted to ensure objectivity and minimize bias.

### PICO-based inclusion and exclusion

The eligibility criteria were structured using the PICO (Population, Intervention, Comparison, Outcome) framework [18] to ensure the inclusion of studies most relevant to the review question. Only those studies that involved human subjects, radiographic results of root resorption, and provided quantification results were included. Animal research, in vitro research, and research with no pertinent comparators or outcomes were eliminated to have a narrow and clinically relevant evidence base (Table 2).

**Table 2:** Eligibility criteria for study selection using the PICO framework.

PICO element	Inclusion criteria	Exclusion criteria
Population	Human subjects undergoing orthodontic treatment	Animal studies, in vitro studies
Intervention	Radiographic assessment of root resorption (2D or 3D methods)	Non-radiographic or biological marker-only studies
Comparison	Within-study orthodontic intervention-versus-comparator contrasts; subgroup comparison by radiographic assessment method (2D vs. 3D/CBCT) and by orthodontic force mechanics (intrusive vs. non-intrusive).	No relevant intervention/comparator contrast or insufficient information to classify radiographic assessment method or orthodontic force mechanics.
Outcome	Quantification of orthodontically induced root resorption (OIRR), reported as linear root length change or volumetric root loss.	Studies not reporting quantitative OIRR outcomes or data sufficient for standardized effect-size extraction.

### Data extraction process

Two reviewers extracted data using a standardized form. Extracted variables were selected to address all three aims and included study design, participant demographics, sample size, orthodontic intervention and comparator, force magnitude when reported, treatment duration, radiographic assessment method, OIRR measurement unit (linear root length change in mm or volumetric root loss in mm³), and numerical data required to calculate standardized effect estimates. Radiographic assessment was classified as 2D imaging (periapical or panoramic radiography) or 3D imaging (CBCT). Orthodontic intervention mechanics were classified as intrusive or nonintrusive after independent review by two reviewers, with disagreements resolved by consensus or consultation with a third reviewer.

### Risk of bias assessment

The tool used to determine the quality of the methodology of the included studies was the Cochrane Risk of Bias-2 (ROB 2) tool of randomized trials [19] and the Risk of Bias in Non-Randomized Studies of Interventions (ROBINS-I) tool of nonrandomized studies [20]. Funnel plots were used to assess publication bias visually and the Egger test statistically to give an idea about the reliability and validity of the synthesized evidence [21].

### Outcome and comparison variables

The sole clinical outcome of this review was quantification of orthodontically induced root resorption (OIRR), measured as either linear root length change (mm) or volumetric root loss (mm³) and converted to correlation coefficients (*r*) for standardized pooling.

The comparison variables were not separate clinical outcomes. They were explanatory variables used to address the review aims: (1) the within-study orthodontic intervention-versus-comparator contrast for the main pooled analysis; (2) radiographic assessment method, categorized as 2D imaging versus 3D/CBCT imaging, for the prespecified subgroup analysis; and (3) orthodontic force mechanics, categorized as intrusive versus nonintrusive, for the post-hoc subgroup analysis.

### Data synthesis and analysis

Random-effects meta-analysis was performed to account for expected clinical and methodological heterogeneity between studies. The primary pooled analysis combined standardized OIRR effect estimates derived from each within-study orthodontic intervention-versus-comparator contrast; therefore, the pooled estimate refers to the average association between the tested orthodontic intervention contrast and the magnitude of OIRR, not to a direct comparison of 2D versus 3D imaging. To harmonize disparate OIRR measures (e.g. root length change in mm and root volume loss in mm³), all effect sizes were converted to the correlation coefficient (*r*). For studies reporting only means and standard deviations, Cohen’s d was calculated and then converted to *r* using the formula *r* = *d* / √(*d*² + 4). Positive r values indicate greater OIRR in the intervention or higher-risk contrast; negative r values indicate less OIRR in that contrast.

To address the radiographic-assessment aim, one prespecified subgroup analysis compared studies using 2D imaging with those using 3D/CBCT imaging. To address the orthodontic-intervention aim and investigate heterogeneity, one post-hoc subgroup analysis compared studies using intrusive mechanics with those using nonintrusive mechanics. Intrusive forces were defined as intentionally apically directed forces used to lower the vertical position of incisors (e.g. intrusion arch or continuous intrusive forces). Alignment, leveling, space closure, or retraction procedures without an intentional apical force component were categorized as nonintrusive mechanics. Between-subgroup differences were tested using analysis of variance (ANOVA), and the proportion of between-study variance explained by each subgroup variable was expressed as pseudo R².

The *I*^2^ statistic was used to determine heterogeneity, and sensitivity analyses were implemented to determine the strength of the results. All statistical analyses were done with Review Manager (RevMan) software version 5.4, and the results were presented in terms of confidence intervals and forest plots.

## Results

### Literature search and screening process

**Figure 1:**
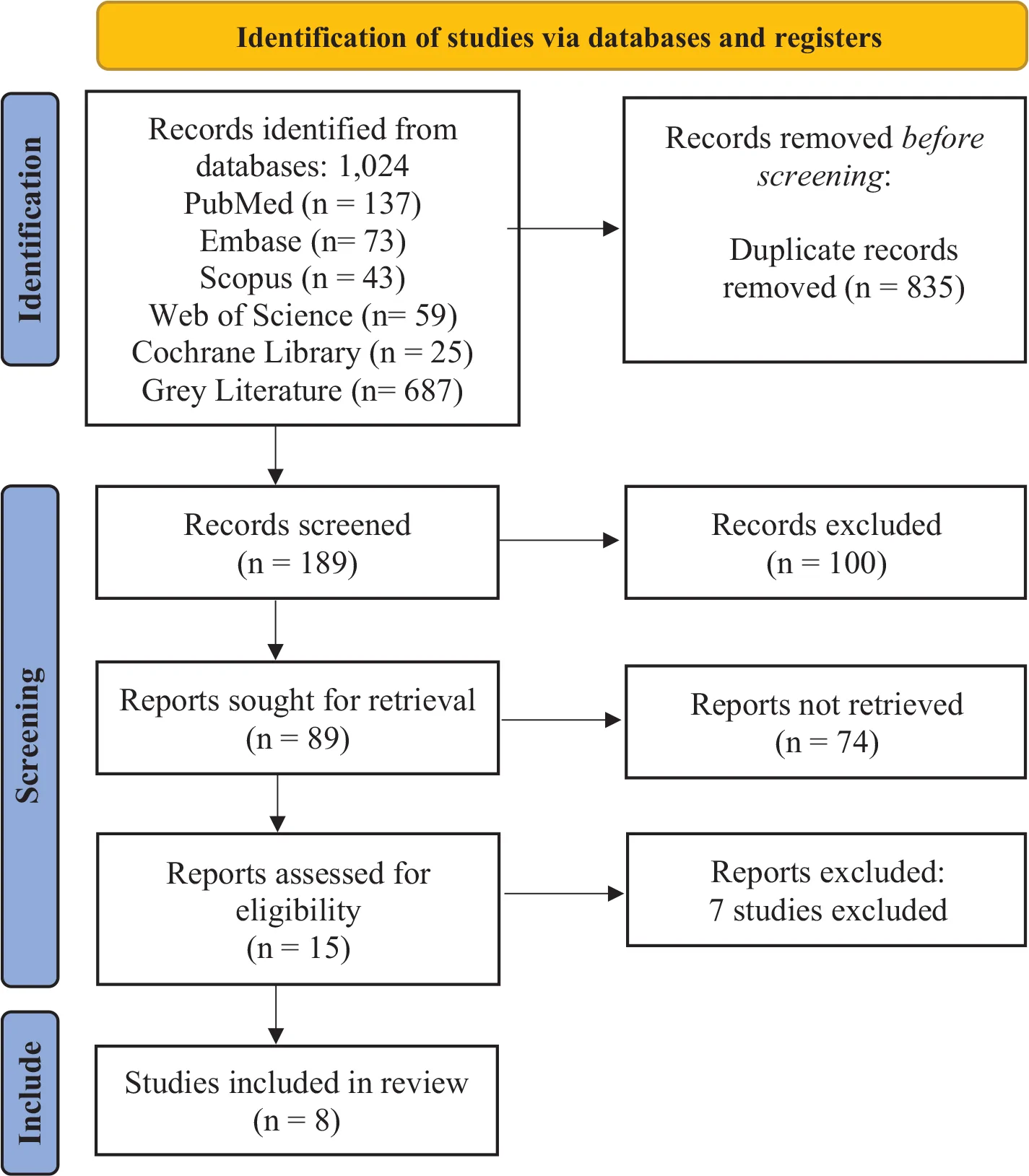
Identification and selection of studies for a systematic review: PRISMA flow diagram.

The systematic review began by identifying a total of 1,024 potential records from several major databases and gray literature sources, with PubMed being the largest contributor. After an initial removal of 835 duplicate records, 189 unique studies were screened for relevance. From these, the full text of 89 reports was sought for detailed evaluation; however, 74 of these could not be retrieved. Ultimately, 15 full-text articles were thoroughly assessed for eligibility against the predefined inclusion criteria. This final stage led to the exclusion of seven studies [22–28] (Table 3), while eight studies were included, finalizing the evidence base for the analysis [29–36] (Figure 1).

**Table 3:** Studies excluded after full-text assessment with reasons.

Study (Author & Year)	Reason for exclusion
Chun et al. (2022) [22]	Focuses on **skeletal and alveolar changes** from palatal expansion, not root resorption. The outcome is not relevant.
Hung et al. (2024) [23]	The population is patients with **impacted third molars** undergoing extraction, not patients undergoing orthodontic treatment.
Salah et al. (2024) [24]	Intervention is **endodontic microsurgery** for periapical lesions. The context is endodontic, not orthodontic.
Attia et al. (2024) [25]	In an orthodontic RCT, the primary outcome is **anchorage loss**, not the detection or quantification of root resorption.
Patel et al. (2025) [26]	Study on **external cervical resorption**; this is an observational study of predisposing features, not an assessment of radiographic accuracy during orthodontic treatment.
Gonzalez-Hernandez et al. (2018) [27]	Completely irrelevant population and intervention. This is a study on **compassion training for breast cancer survivors**.
Pul et al. (2025) [28]	Focuses on AI diagnosis of **periapical radiolucencies** (endodontic lesions), not orthodontically induced root resorption.

### Characteristics of the included studies

Table 4 revealed a robust evidence base, primarily composed of randomized controlled trials (RCTs) that explore a variety of orthodontic interventions – from different archwire types and force levels to adjunctive therapies like vibration and micro-osteoperforations (MOPs). The main one is that there is a general trend to use CBCT in subsequent studies that allow the transition of the simple linear measurements of 2D radiographs to the accurate and three-dimensional quantification of the root structure loss in volumetric terms, which gives better data to analyze.

**Table 4:** Summary table of included studies on orthodontically induced root resorption.

Study (Author, Year)	Study design	Participant demographics	Orthodontic intervention	Radiographic method	Diagnostic outcome for root resorption
Alzahawi et al. (2014) [29]	Prospective study	82 patients	Leveling with super-elastic NiTi vs. conventional steel archwires	Periapical radiographs	Quantification of root resorption (length)
Karadeniz et al. (2013) [30]	Randomized controlled trial	48 patients	Heavy (225 g) vs. light (25 g) orthodontic force application	Panoramic and periapical radiographs	Quantitative measurement of root resorption (volume using VRF software)
de Almeida et al. (2018) [31]	Randomized controlled trial	28 patients	Intrusion arch mechanics vs. straight wire mechanics	CBCT	Quantification of root volume loss
DiBiase et al. (2016) [32]	Multicenter randomized controlled trial	72 patients	Supplemental vibrational force (AcceleDent) vs. no vibration during alignment	CBCT	Quantitative measurement of root resorption (volume)
Phermsang-Ngarm et al. (2018) [33]	Randomized controlled trial	32 adult patients	Initial alignment with preformed NiTi vs. customized NiTi archwires	CBCT	Quantification of root resorption (volume and length)
Raza et al. (2016) [34]	Prospective, double-blind, controlled trial	10 patients	LIPUS vs. sham device during torque application	CBCT	Quantitative measurement of root resorption (volume)
Mordente et al. (2024) [35]	Randomized controlled trial	37 patients	MOPs vs. no MOPs during incisor retraction	CBCT	Quantification of root resorption (volume) as a secondary outcome
Shaadouh et al. (2025) [36]	Randomized controlled trial	34 young adults	LIES vs. sham during en masse retraction	CBCT	Quantitative measurement of EARR

NiTi: Nickel Titanium; g: grams; VRF: volumetric root formula (or the specific software used for volumetric measurement); CBCT: cone-beam computed tomography; MOPs: micro-osteoperforations; LIPUS: low-intensity pulsed ultrasound; LIES: low-intensity electrical stimulation; EARR: external apical root resorption.

The evidence synthesized in the eight studies involved gives a multidimensional interaction between orthodontic interventions and external apical root resorption (EARR). Mechanical-based components of treatment were always found to be a major contributor to treatment. An example is a study by Alzahawi et al. [29] and Phormsang-Ngarm et al. [33], which examined the type of archwire used, which found that the first phase of the process of archwire arch alignment, regardless of whether it is super-elastic or conventional archwire, is always associated with measurable shortening of the root, and that suggests that some amount of resorption is an inherent risk of active tooth movement. It was also underlined by de Almeida et al. [31], who showed that more vigorous mechanics, namely intrusion arch therapy, resulted in a significantly higher degree of incisor root volume loss than straight-wire mechanics, which illustrates the extent to which a particular treatment modality poses a certain risk.

Some studies were conducted in reaction to this identified risk to empirically assess adjunctive treatments aimed at alleviating EARR. One of the most noticeable results of all these trials was the overall inefficiency of such devices. Supplemental vibrational force, as examined by DiBiase et al. in a large multicenter trial [32], and low-intensity pulsed ultrasound (LIPUS), as examined by Raza et al. [34], both found that the interventions did not significantly reduce root resorption in both cases as compared to their respective control groups. On the same note, MOPs to speed up the movement of the teeth, studied by Mordente et al. [35], and the use of low-intensity electrical stimulation (LIES) by Shaadouh et al. [36] did not show any protective effect of the roots during space closure and retraction. All this evidence indicates that although these techniques can have an effect on the speed of tooth movement, they do not seem to provide a clinically significant effect in the maintenance of root structure.

Unlike such mechanical and apparatus-driven methods, the article by Karadeniz et al. proposed an important biological variable [30]. In their study, they found that the root resorption caused by the use of heavy continuous force was predictable, but the concurrent administration of fluoride possessed a significant protective action, which lowered the resorption craters in the high-force group by a significant margin. This observation is important in suggesting that mechanical forces can meaningfully interact with chemical or biological cofactors to affect the resorptive process, which is a potential avenue of preventive measures, unlike physical adjuncts.

Finally, based on all the evidence, orthodontically induced inflammatory resorption of the root is a direct and measurable treatment outcome. The risk is also dependent on the selection of mechanics, where the more intrusive the forces, the more the root is lost. Nevertheless, as the existing evidence indicates, the effectiveness of some of the current trendy physical adjunctive interventions, such as vibrational devices, LIPUS, MOPs and LIES, in the prevention of this unwanted effect is poorly supported, indicating the existence of a critical gap between their hypothetical positive effects and clinical fact. The possible application of biochemical substances such as fluoride, however, is subject to research.

### Risk of bias assessment for included studies

#### Risk of bias

**Figure 2:**
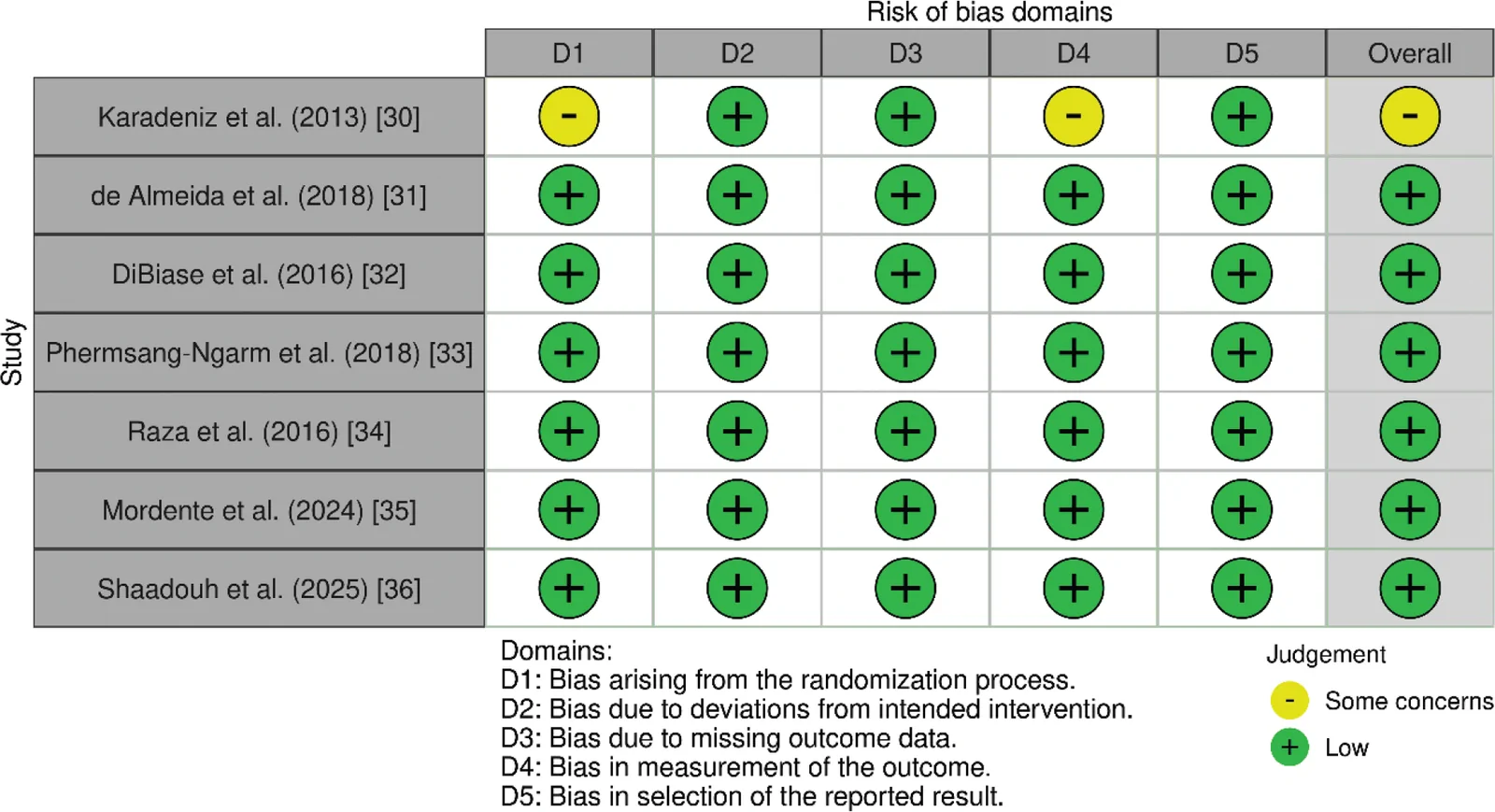
Risk of bias summary for included randomized controlled trials (RoB 2 Tool).

**Figure 3:**
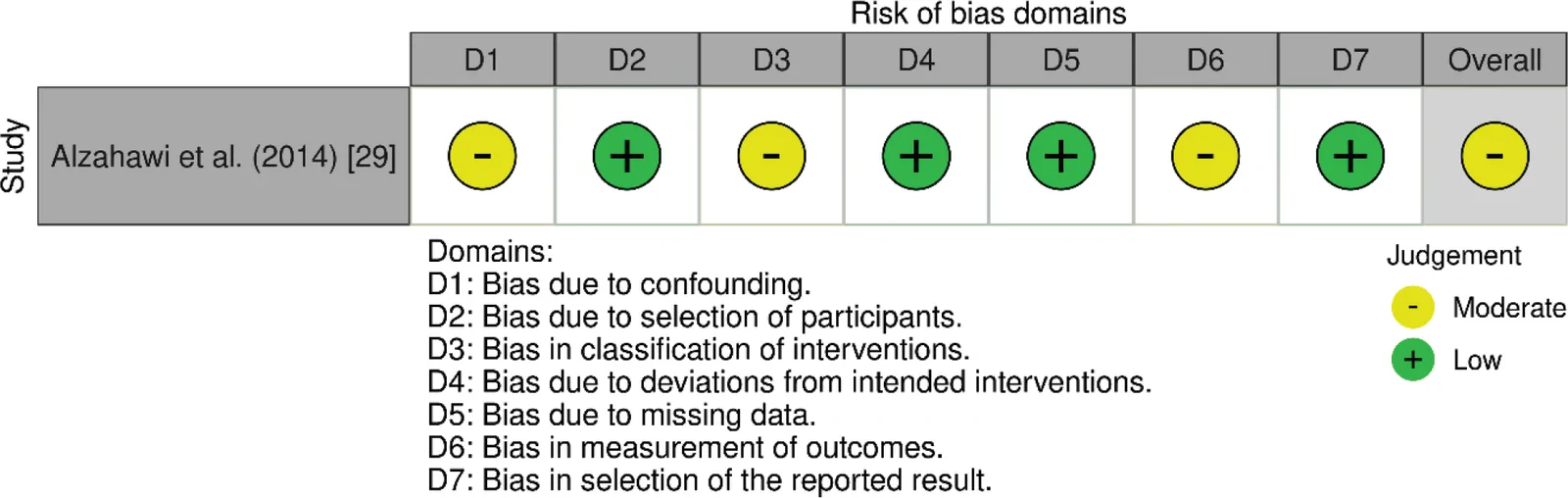
Risk of bias assessment for the nonrandomized study (ROBINS-I Tool).

The risk of bias evaluation showed that the included RCTs possess a high level of methodological rigor, as six out of seven RCTs presented a low risk of bias. One RCT [30] raised some concerns due to a lack of blinding in outcome assessment (Figure 2). The sole nonrandomized study [29] was judged to have a moderate risk of bias due to potential confounding, classification of intervention, and measurement of outcomes (Figure 3).

#### Publication bias

**Figure 4:**
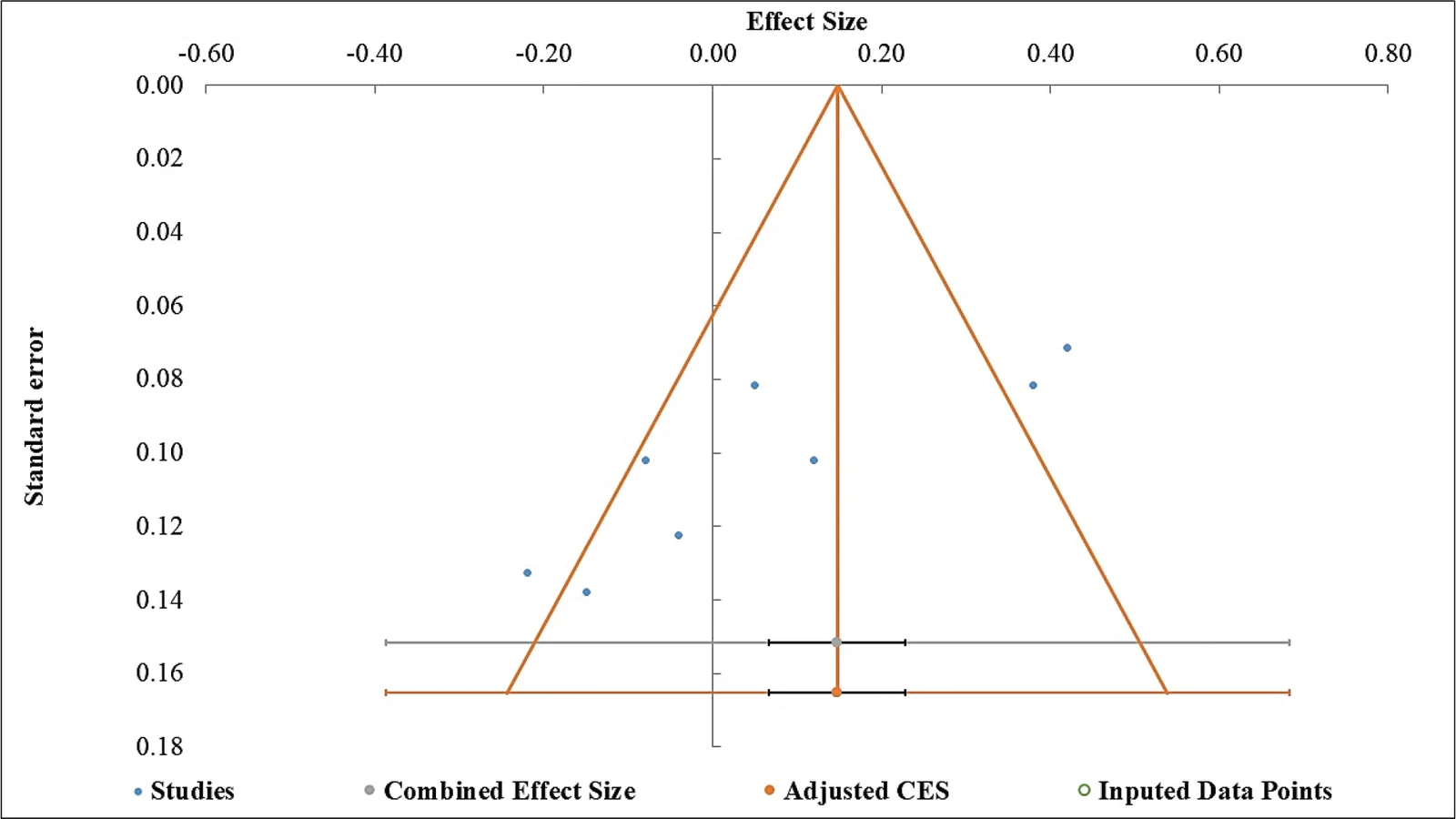
Funnel plot for assessment of publication bias.

A funnel plot indicated an asymmetrical spread (Figure 4), and there were relatively few studies found in the lower-left quadrant, which could indicate that small studies that were reporting null or negative effects may be missing. The Egger linear regression yielded an intercept of −8.79 (95% CI: −14.31 to −3.28) and a slope of 0.97 (95% CI: 0.44 to 1.50). The *t*-value of the intercept was −3.77 with a *p*-value of 0.009, which is statistically significant at the traditional alpha level of 0.05. The true heterogeneity of the effect sizes (e.g. the variation in the quality of studies, type of interventions, or measures of outcomes) may also lead to asymmetry. However, the significant Egger’s test should be approached with caution to interpret the pooled effect estimate because the published literature can have an overrepresentation of studies with positive or significant results [37, 38] (Table 5).

**Table 5:** Egger’s regression test for funnel plot asymmetry.

Parameter	Estimate	Standard error	95% confidence interval-lower limit	95% confidence interval-upper limit
Intercept	−8.79	2.33	−14.31	−3.28
Slope	0.97	0.22	0.44	1.50
*t*-value	−3.77			
*p*-value	0.009			

### Primary pooled analysis and heterogeneity assessment

**Figure 5:**
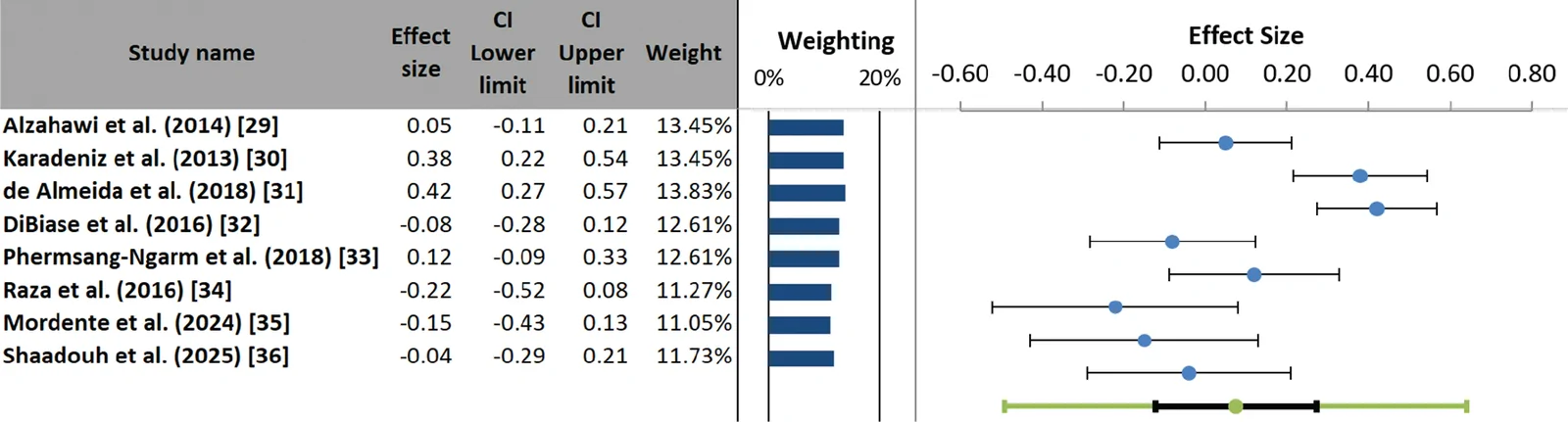
Forest plot of individual study effect sizes and pooled estimate for orthodontic root resorption.

The primary random-effects meta-analysis pooled eight within-study orthodontic intervention-versus-comparator contrasts after standardization to correlation coefficients. This main pooled estimate therefore represents the average OIRR effect associated with the tested orthodontic intervention contrasts across the included clinical trials. The pooled effect size was small and nonsignificant (*r* = 0.07; 95% CI: −0.12 to 0.27; *p* = 0.372). The prediction interval ranged from −0.49 to 0.64, indicating uncertainty regarding the likely range of effects in future studies. Heterogeneity was substantial (Cochran *Q* = 43.84, *p* < 0.001; *I*² = 84.03%), and between-study variance was evident (τ² = 0.05; τ = 0.22). These findings indicate that OIRR effects are context specific and may vary according to mechanical, biological, and methodological factors (Figure 5 and Table 6) [39].

**Table 6:** Pooled effect estimate and heterogeneity analysis from random-effects meta-analysis.

Meta-analysis	Value
Model	Random-effects model
Confidence level	95%
Effect size (correlation)	0.07
Standard error	0.08
Confidence interval, lower limit	−0.12
Confidence interval, upper limit	0.27
Prediction interval, lower limit	−0.49
Prediction interval, upper limit	0.64
*Z*-value	0.89
One-tailed *p-*value	0.186
Two-tailed *p*-value	0.372
Number of included studies	8
Heterogeneity statistics	
*Q* (Cochran’s)	43.84
*pQ*	0.000
*I*²	84.03%
T² (tau-squared)	0.05
T (tau)	0.22

### Subgroup analysis

**Figure 6:**
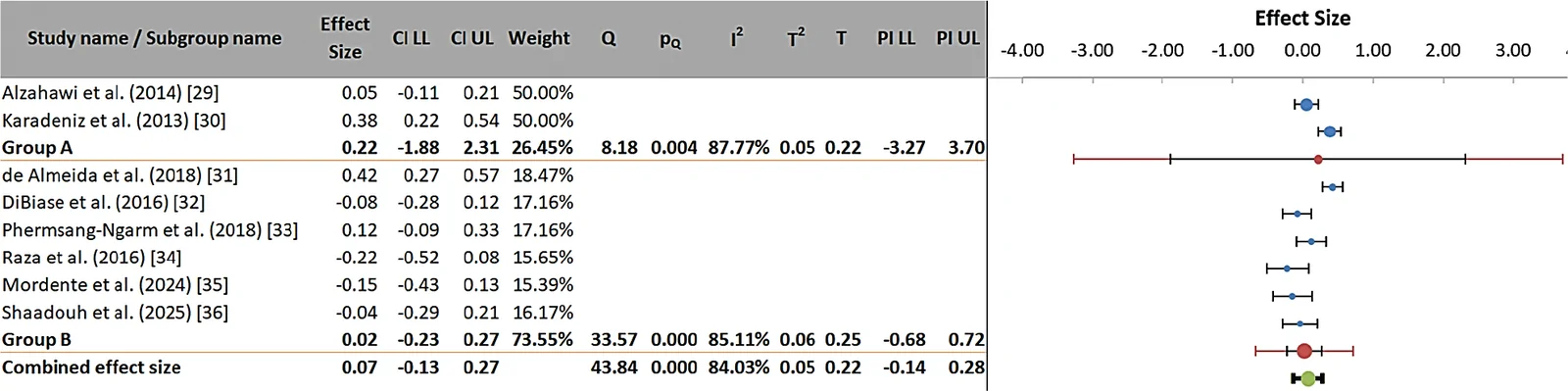
Subgroup analysis forest plot by radiographic modality (2D vs. 3D).

For the radiographic-assessment aim, the prespecified subgroup analysis compared studies using 2D imaging (periapical or panoramic radiography; two studies) with studies using 3D/CBCT imaging (six studies). The pooled effect size for 2D imaging was *r* = 0.22 (95% CI: −1.88 to 2.31), whereas the pooled effect size for 3D/CBCT imaging was *r* = 0.02 (95% CI: −0.23 to 0.27). Within-group heterogeneity remained high in both subgroups (2D: *I*² = 87.85%; 3D/CBCT: *I*² = 85.1%). The between-subgroup test was not statistically significant (Q* = 0.95, df = 1, *p* = 0.331), and radiographic assessment method explained only 16.8% of the between-study variance. These results indicate that the choice of 2D versus 3D/CBCT imaging did not systematically change the direction or magnitude of the pooled OIRR effect estimate (Figure 6 and Table 7).

**Table 7:** Subgroup meta-analysis results and between-group differences.

Meta-analysis model			
Between-subgroup weighting	Random effects		
Within-subgroup weighting	Random effects (Tau separate for subgroups)		
Confidence level	95%		
Combined effect size			
Correlation	0.07		
Standard error	0.09		
Confidence interval, lower limit	−0.13		
Confidence interval, upper limit	0.27		
Prediction interval, lower limit	−0.14		
Prediction interval, upper limit	0.28		
Number of included observations	343		
Number of included studies	8		
Number of subgroups	2		
Analysis of variance	Sum of squares (Q*)	df	*p*
Between / model	0.95	1	0.331
Within / residual	4.67	6	0.586
Total	5.62	7	0.585
Pseudo *R*^2^	16.82%		

**Figure 7:**
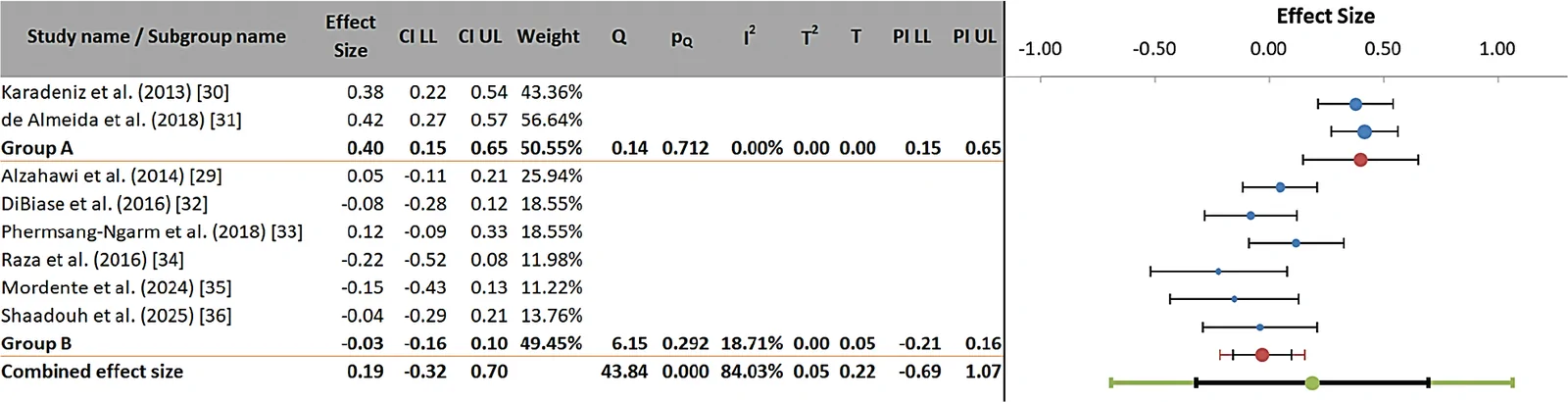
Subgroup analysis forest plot by force type (intrusive vs. non-intrusive).

For the orthodontic-intervention aim, the post-hoc subgroup analysis compared intrusive with nonintrusive force mechanics. The pooled effect size for intrusive mechanics was *r* = 0.40 (95% CI: 0.15 to 0.65), indicating a moderate positive association with increased OIRR. In contrast, the pooled effect size for nonintrusive mechanics was approximately zero (*r* = −0.03; 95% CI: −0.16 to 0.10), indicating no consistent association with OIRR. The between-subgroup difference was statistically significant (Q* = 34.87, df = 1, *p* < 0.001), and force mechanics explained 87.11% of the between-study variance. This finding suggests that intrusive force mechanics are a major contributor to OIRR heterogeneity, whereas nonintrusive alignment, leveling, space-closure, or retraction mechanics were associated with negligible average OIRR effects (Figure 7 and Table 8).

**Table 8:** Subgroup meta-analysis results and between-group differences for force type.

Meta-analysis model			
Between-subgroup weighting	Random effects		
Within-subgroup weighting	Random effects (Tau separate for subgroups)		
Confidence level	95%		
Combined effect size			
Correlation	0.19		
Standard error	0.22		
Confidence interval, lower limit	−0.32		
Confidence interval, upper limit	0.70		
Prediction interval, lower limit	−0.69		
Prediction interval, upper limit	1.07		
Number of included observations	343		
Number of included studies	8		
Number of subgroups	2		
Analysis of variance	Sum of squares (Q*)	df	*p*
Between / model	34.87	1	0.000
Within / residual	5.16	6	0.524
Total	40.03	7	0.000
Pseudo *R*^2^	87.11%		

## Discussion

The primary random-effects meta-analysis showed that, when the included clinical trials were pooled as orthodontic intervention-versus-comparator contrasts, the average OIRR effect was small and not statistically significant (*r* = 0.07; 95% CI: −0.12 to 0.27). This result should not be interpreted as evidence that all orthodontic interventions carry the same OIRR risk. Instead, the substantial heterogeneity indicates that OIRR magnitude is strongly context dependent, particularly on the basis of the applied force system. Our findings are consistent with previous evidence showing that intrusive mechanics concentrate stress near the root apex and are associated with greater resorption risk [8].

While three-dimensional imaging, specifically CBCT, offers superior volumetric data, its use must be justified against the ALARA (as low as reasonably achievable) principle. Effective doses for CBCT range from 5–50 µSv for small-field protocols to over 100 µSv for large-field scans, compared to 2–5 µSv for periapical radiographs [40, 41]. Current guidelines recommend CBCT only when 2D imaging cannot adequately answer the clinical question (e.g. in cases of suspected severe root resorption or before orthognathic surgery), rather than as a routine monitoring tool for all patients [42].

Hajeer et al. found superior diagnostic performance of CBCT over conventional radiography in localization tasks, supporting the view that 3D (CBCT) imaging reduces interpretative uncertainty [43]. Alsino et al. validated CBCT’s higher sensitivity for detecting cortical defects, a finding that parallels CBCT’s improved detection of buccal/lingual root resorption compared with 2D imaging [44]. Sirri et al. used CBCT to quantify root resorption after minimally invasive acceleration procedures, illustrating how 3D (CBCT) imaging enables more reliable secondary outcome assessment in clinical trials [45].

With respect to the radiographic-assessment aim, the subgroup analysis found no statistically significant difference between pooled OIRR estimates derived from 2D imaging and those derived from 3D/CBCT imaging (*p* = 0.331). Thus, although CBCT improves anatomical visualization and volumetric quantification, the imaging modality itself did not explain most of the variability in reported OIRR effects in this meta-analysis.

Substantial heterogeneity persisted within both radiographic subgroups (*I*² > 85%), indicating that variables beyond imaging technology, including orthodontic mechanics, force magnitude, treatment duration, and patient-specific biological responses, are likely to be more influential determinants of effect-size variability [46, 47]. Therefore, CBCT should be regarded as a more accurate assessment tool for selected clinical and research scenarios, rather than as an explanation for differences in intervention-related OIRR effects.

The possible protective effect of systemic fluoride observed in a single small study [30] suggests a potential biological mechanism worthy of further investigation. However, given the limited evidence (one study, *n* = 48), no clinical recommendations can be made, and this finding should be considered hypothesis-generating only. This result can indicate that a more effective strategy could be to increase the resistance of the root to osteoclastic activity on a biochemical level rather than to change the physical stimulus. This effect of fluoride could be explained by the established property of increasing mineral density and solubility of fluoride, especially in high-force circumstances. Although it was one of the studies that gave this finding, a new set of preventive strategies aimed at the molecular pathways of resorption is being pointed out in this finding, though it needs additional validation in the form of special RCTs.

With respect to the orthodontic-intervention aim, force mechanics was the most important identified source of between-study heterogeneity. The post-hoc subgroup analysis showed a highly significant difference between intrusive and nonintrusive mechanics (*p* < 0.001). Intrusive mechanics showed a moderate positive association with OIRR (*r* = 0.40; 95% CI: 0.15 to 0.65), whereas nonintrusive mechanics showed no consistent association (*r* = −0.03; 95% CI: −0.16 to 0.10). The pseudo R² of 87.1% suggests that force mechanics explained most of the between-study variance observed in this review. This observation is consistent with prior studies showing that intrusive forces localize mechanical stress at the root apex and can initiate focal inflammatory resorption [8, 48].

Notably, the minimal heterogeneity in the intrusive subgroup (*I*^2^ = 0.0) indicates that the association between intrusion and root resorption can be shaped and replicated using different study groups and protocols. The clinical implications of these findings are that the extent to which root resorption can be inevitable during orthodontic treatment is moderate, but there is a significantly increased risk when intrusive mechanics are involved. On the other hand, procedures of alignment and retraction that do not involve intentional intrusion seem to be of low risk of showing clinically significant root resorption on average.

This review is limited in a number of ways, notwithstanding the rigorous methodology. The most notable of them is the high rate of statistical heterogeneity among the included studies, which makes it more difficult to interpret the pooled effect estimate. This nonhomogeneity is perhaps due to the differences in clinical and methodological philosophy, such as differences in patient age, treatment plans, specific orthodontic mechanisms, and even the exact ways of analyzing the CBCT. The limited number of studies that could be used to conduct certain comparisons also did not allow for the power of the subgroup analyses. The high Egger test (*p* = 0.009) statistically indicates the presence of funnel plot asymmetry, and it may be possible that there is publication bias. A nonsignificant or negative result of smaller studies that involve root resorption might not be published, which will inflate the pooled effect estimate. Asymmetry may, however, also be due to true between-study heterogeneity, which was high in this meta-analysis (*I*^2^ = 84.0%). The pooled estimate should thus be construed with caution by the readers.

Another strength of this systematic review is its prospective registration in PROSPERO (CRD420251245978). Registration increases transparency, reduces the risk of reporting bias, and allows readers to compare planned versus conducted analyses. However, some limitations are worth considering. First, although the review was registered, the PROSPERO record was completed after the literature search had started, which may create some risk of selective outcome reporting. Second, the force-type subgroup analysis was not prespecified in the PROSPERO registration and is therefore clearly interpreted as a post-hoc, hypothesis-generating analysis rather than a confirmatory finding. Third, a number of full-text articles could not be retrieved (74 of 89 sought, 83.1%). Most unrecovered records were gray literature sources, conference abstracts, unpublished theses, or institutional reports without accessible full-text versions. This may create selection bias, particularly if unpublished or nonpeer-reviewed studies with null or negative findings were systematically excluded. Because all eight included studies were published and peer reviewed, the pooled estimate may overrepresent positive or significant findings.

Because OIRR was the only clinical outcome, conversion of heterogeneous OIRR measurement units (linear root length change in mm and volumetric root loss in mm³) to a common metric (correlation coefficient r) was necessary for meta-analysis. However, this transformation may obscure clinically meaningful differences between measurement types. For example, a 0.5 mm linear shortening may not be clinically equivalent to a 10 mm³ volume loss, although both can be transformed to the same r metric. Future studies should report standardized OIRR units and provide sufficient data to support direct comparisons.

The small number of studies available for subgroup analyses (*n* = 2 for 2D imaging; *n* = 6 for 3D imaging) limits statistical power and precludes meta-regression or more nuanced exploration of heterogeneity sources. The wide confidence intervals for the 2D subgroup effect estimate (95% CI: −1.88 to 2.31) reflect this imprecision, and findings from subgroup comparisons should be interpreted cautiously.

Confounding variables such as exact force magnitude (grams), treatment duration (months), patient age, and genetic predisposition could not be quantitatively assessed due to inconsistent reporting across studies. These factors may significantly influence OIRR severity and could account for some of the residual heterogeneity not explained by force type or imaging modality.

Further studies are required to focus more on well-designed multicenter RCTs that have standardized protocols to reduce heterogeneity and further conclude definitively. Such studies are to specifically examine how much biochemical agents such as fluoride will be promising in the prevention of OIRR in comparison to placebo controls. Longitudinal studies that would follow root resorption post-retention period through pretreatment, with consistent 3D volumetric analysis, are also required to learn the ultimate destiny of the resorbed roots. Lastly, the incorporation into the program of an emerging technology like artificial intelligence to automate root segmentation on CBCT scans may not only improve the precision and efficacy of the measurement but also enable the detection of subtle patterns and risk factors, which are currently impossible to detect, and bring true individualism in the risk analysis during the orthodontic treatment planning process.

In conclusion, this systematic review and meta-analysis showed that the overall pooled OIRR effect across orthodontic intervention-versus-comparator contrasts was small and nonsignificant. The radiographic assessment method did not significantly alter the pooled estimate, although 3D/CBCT imaging provides superior volumetric quantification and is valuable for research and selected high-risk clinical cases when justified under the ALARA principle. The magnitude of OIRR was most strongly influenced by orthodontic force mechanics: intrusive mechanics showed a moderate association with increased resorption, whereas nonintrusive mechanics showed no consistent association. Because the force-type finding was based on a post-hoc subgroup analysis, it should be interpreted as hypothesis-generating, but it emphasizes the clinical need for careful force selection, selective radiographic monitoring, and further well-designed trials using standardized OIRR measurement methods.
